# The Improvement of Cardiac and Endothelial Functions of Xue-Fu-Zhu-Yu Decoction for Patients with Acute Coronary Syndrome: A Meta-Analysis of Randomized Controlled Trials

**DOI:** 10.1155/2022/2671343

**Published:** 2022-02-10

**Authors:** Shiqi Chen, Xiaoxiao Wu, Tong Li, Weiting Cheng, Xiaowan Han, Yang Li, Baofu Wang, Yu Teng, Mingjing Zhao, Yahong Wang

**Affiliations:** ^1^Department of Cardiology, Dongzhimen Hospital Affiliated to Beijing University of Chinese Medicine, Beijing 100700, China; ^2^Key Laboratory of Chinese Medicine of Ministry of Education and Beijing, Dongzhimen Hospital Affiliated to Beijing University of Chinese Medicine, Beijing 100700, China

## Abstract

**Background:**

Xue-Fu-Zhu-Yu decoction (XFZYD) is a traditional Chinese prescription that has been used to treat patients with blood stasis in China for many years. The present study aimed to evaluate the improvement of cardiac and endothelial functions of XFZYD for patients with acute coronary syndrome (ACS) through a systematic review and meta-analysis.

**Methods:**

Six databases were searched to collect RCTs related to the treatment of XFZYD for ACS. The primary outcomes were cardiac and endothelial functions, including the levels of left ventricular ejection fraction (LVEF), left ventricular end-diastolic diameter (LVEDD), and left ventricular end-systolic diameter (LVESD) in echocardiography, as well as the changes in the levels of nitric oxide (NO), endothelin-1 (ET-1), intercellular adhesion molecule-1 (ICAM-1), and vascular cell adhesion molecule-1 (VCAM-1) in the serum. The secondary outcomes were the blood levels of oxidative damage markers (including superoxide dismutase (SOD) and malondialdehyde (MDA)), C-reactive protein (CRP), brain natriuretic peptide (BNP), creatine kinase-MB (CK-MB), and cardiac troponin I (cTnI) as well as the incidence of adverse drug reactions (ADRs). Weighted mean difference (WMD) was estimated for all the outcomes with the random effects model. This type of analysis was conducted in the subgroups of the ACS subtypes, and the methodological quality was assessed using the handbook of Cochrane Collaboration.

**Results:**

A total of 1,658 records were identified, and 16 randomized controlled trials (1,171 patients) were included. The primary outcomes suggested that XFZYD combined with routine treatment improved LVEF, reduced LVEDD and LVESD, and also improved the serum levels of NO, and reduced the levels of ET-1 and ICAM-1. XFZYD combination therapy significantly ameliorated the blood levels of SOD, MDA, BNP, CK-MB, and cTnI. However, the results indicated no significant difference between XFZYD plus routine treatment and routine treatment for the levels of VCAM-1 and CRP. Moreover, all the ADRs reported in the included studies were slight and the patients recovered soon.

**Conclusions:**

The present study suggested that XFZYD may improve the cardiac and endothelial functions of ACS patients without serious ADRs. However, based on the mediocre methodological quality, the aforementioned conclusion should be confirmed in a multicenter, large-scale, and accurately designed clinical trial.

## 1. Introduction

Coronary heart disease (CHD) has been a leading cause of the incidence and mortality worldwide, and it can be divided into acute coronary diseases and stable angina pectoris [1, 2]. Acute coronary syndrome (ACS), which contains three conditions, including ST-elevation myocardial infarction (STEMI), non-ST-elevation myocardial infarction (NSTEMI), and unstable angina (UA), can obstruct the balance of myocardial oxygen supply and demand and result in myocardial ischemia [3]. Untreated UA (without serologic elevation of troponin or creatine kinase-MB (CK-MB) isoenzyme concentration) may dynamically progress to myocardial infarction (MI); both NSTEMI and STEMI, which can be distinguished by the ST-segment elevation in the electrocardiogram (ECG), exhibit serological evidence of myonecrosis [3]. ACS is mainly caused by the rupture of vulnerable atherosclerotic coronary plaques or the acute formation of the thrombus. Arterial thrombosis due to superficial erosion can activate the endothelium leading to endothelial apoptosis and detachment and induce inflammation and oxidative stress, which affects endothelial dysfunction and platelet aggregation [4, 5]. Transient or long-term myocardial ischemia also results in impaired cardiac function. Recent studies have shown that the western therapies for ACS are related to inhibition of angina, platelet aggregation, and thrombosis, lowering of cholesterol levels by statin medications, as well as percutaneous coronary intervention (PCI) surgery [3, 6]. However, these therapies may be inappropriate for certain individuals and can produce specific adverse drug reactions (ADRs) [7–9]. Traditional Chinese medicine (TCM) plays a more extensive therapeutic role in patients who do not undergo PCI surgery and improve the functions of the heart and blood vessels. Under these circumstances, remedies of complementary and alternative medicine are used so as to enhance the therapeutic effect and reduce adverse reaction-associated toxicity.

Xue-Fu-Zhu-Yu decoction (XFZYD) is a well-known TCM formula, which was originally founded by the famous TCM doctor Qingren Wang from the Qing dynasty. XFZYD consists of the following 11 Chinese herbs: *Angelicae sinensis* (Oliv.), Diels (Danggui), *Rehmannia glutinosa* Libosch. (Dihuang), *Prunus persica* (L.) Batsch (Taoren), *Carthamus tinctorius* L. (Honghua), *Glycyrrhiza uralensis* Fisch. (Gancao), Aurantii Fructus (Zhiqiao), *Paeonia lactiflora* Pall. (Chishao), *Bupleurum chinense* DC. (Chaihu), *Ligusticumi chuanxiong* Hort. (Chuanxiong), *Platycodon grandiflorum* (Jacq.) A. DC. (Jiegeng), and *Achyranthes bidentata* Bl. (Niuxi). The decoction contains at least 34 major constituents including 21 flavonoids, 5 terpenoids, 3 organic acids, 2 lactones, 1 alkaloid, 1 amino acid, and 1 cyanogenic glycoside, which play important roles in the prevention of various cardiovascular diseases [10]. XFZYD has been identified to treat UA patients with blood stasis and Qi stagnation symptoms [11, 12], and a variety of its dosage forms have also been approved by the National Medical Products Administration of the Chinese government for clinical practice. Previous studies [13–15] have shown that the integration of XFZYD and Western medicines can improve the angina symptoms and the blood levels of lipids in patients with CHD. However, the amelioration of the cardiac and endothelial functions due to the combination of XFZYD with routine treatment (RT) in ACS has not been fully explored. The indicators detecting these functions are objective in nature and the results are beneficial to assess the authentic efficacy of XFZYD. Considering that a number of clinical trials have examined the use of XFZYD to treat ACS in recent years, we sought to evaluate its efficacy in improving cardiac and endothelial functions through a systematic review and meta-analysis.

## 2. Materials and Methods

This review protocol was registered on PROSPERO (CRD 42021253651).

### 2.1. Criteria for considering the Studies in This Review

#### 2.1.1. Types of Studies

Randomized controlled trials (RCTs) with no limit of publishing language were included.

#### 2.1.2. Types of Participants

The patients that were identified according to accurate ACS diagnostic criteria, which were similar to previously published guidelines [16, 17], were eligible for inclusion in this study. According to the International Classification of Diseases 10th revision (ICD-10) codes, the patients with STEMI, NSTEMI, or UA with or without PCI could be included in the present study [1, 18].

#### 2.1.3. Types of Interventions in the Experimental and Control Groups

The intervention in the experimental group included XFZYD without addition or subtraction of any herbs and should be combined with the routine treatment (RT) of the control group. The intervention in the control group was the conventional therapy without any other traditional Chinese medicines. RT should be the regular medications of inhibition of angina, platelet aggregation, thrombosis, arrhythmia, hypertension, and diabetes as well as statins according to the guidelines [3, 6].

#### 2.1.4. Types of Outcome Measures

The primary outcomes were defined as measures of cardiac and endothelial functions. The levels of left ventricular ejection fraction (LVEF), left ventricular end-diastolic diameter (LVEDD), and left ventricular end-systolic diameter (LVESD) in the echocardiography measurement were used to identify the cardiac function, and the changes in the levels of nitric oxide (NO), endothelin-1 (ET-1), intercellular adhesion molecule-1 (ICAM-1), and vascular cell adhesion molecule-1 (VCAM-1) in the serum were representative of the endothelial function. The additional outcomes observed included the blood levels of the oxidative damage markers (superoxide dismutase (SOD) and malondialdehyde (MDA)), C-reactive protein (CRP), brain natriuretic peptide (BNP), and the myocardial enzymes (CK-MB and cardiac troponin I (cTnI)) and ADRs.

### 2.2. Information Sources and Search Strategy

The search was applied to the following six databases: Cochrane Library, PubMed, Web of Science, the Chinese National Knowledge Infrastructure (CNKI), Wanfang Database, and Weipu Database (VIP), from the inception of each electronic database to February 15, 2021. Additional identification was conducted for all eligible trials by other searching methods from websites and citations. The following terms were used as the mesh terms or the free terms: “acute coronary syndrome,” “ACS,” “myocardial infarction,” “MI,” “ST-segment elevation myocardial infarction,” “STEMI,” “non-ST-segment elevation myocardial infarction,” “NSTEMI,” “unstable angina,” “UA,” “unstable angina pectoris,” “UAP,” “Xuefuzhuyu,” “xue-fu-zhu-yu,” “xuefuzhuyu,” and “xue fu zhuyu.” The searching strategy in PubMed was performed as (“acute coronary syndrome”[MeSH Terms] OR “acute coronary syndrome”[All Fields] OR “ACS”[All Fields] OR “myocardial infarction”[MeSH Terms] OR “myocardial infarction”[All Fields] OR “ST elevation myocardial infarction”[MeSH Terms] OR “ST elevation myocardial infarction”[All Fields] OR “ST segment elevation myocardial infarction”[All Fields] OR “STEMI”[All Fields] OR “non ST elevated myocardial infarction”[MeSH Terms] OR “non ST elevated myocardial infarction”[All Fields] OR “NSTEMI”[All Fields] OR “angina, unstable”[MeSH Terms] OR (“angina”[All Fields] AND “unstable”[All Fields]) OR “unstable angina”[All Fields] OR (“unstable”[All Fields] AND “angina”[All Fields] AND “pectoris”[All Fields]) OR “unstable angina pectoris”[All Fields] OR “UAP”[All Fields] OR “UA”[All Fields]) AND (“Xuefuzhuyu”[All Fields] OR “xue-fu-zhu-yu”[All Fields] OR “xue fu zhu yu”[All Fields] OR “xuefu zhuyu”[All Fields]).

### 2.3. Study Selection

Two investigators (SQC and TL) independently carried out a literature search using the predetermined criteria in the NoteExpress 3.2 software. Initially, duplications were found in all databases and removed from the original search results. Furthermore, the apparently irrelevant studies were excluded following the reading of the titles and abstracts. Finally, the full texts were screened to identify the related studies, and the unqualified studies were excluded. The process of study selection was cross-checked by two researchers. Any disagreement was discussed and resolved in a consensus meeting with the corresponding author (YHW and MJZ).

### 2.4. Data Extraction

After the selection, two authors (XXW and WTC) independently extracted data from the included studies by using a standardized sheet, which was prepared for this review. The extracted data included the study title, name of the first author, year of publication, the diagnostic criteria of the patients, age of participants, baseline, disease types, sample size, interventions in the treatment and control groups, treatment duration, treatment dose, and outcome measures.

### 2.5. Risk of Bias in Individual Studies

Two authors (XWH and YL) independently assessed the risk of bias using assessment tools provided by the handbook of Cochrane Collaboration to evaluate the methodological quality of included studies, involving the blinding of outcomes assessment (i.e., detection bias), the blinding of participants and personnel (i.e., performance bias), the random sequence generation (i.e., selection bias), the allocation concealment (i.e., selection bias), the incomplete outcomes data (i.e., attrition bias), the selective reporting (i.e., reporting bias), and other biases. Disagreements were resolved by consensus with the corresponding author (YHW and MJZ).

### 2.6. Strategy for Data Synthesis

#### 2.6.1. Statistical Analysis

Review Manager 5.3 software provided by the Cochrane Collaboration was used to conduct data analysis. Dichotomous data were calculated as the risk ratios (RR) and the 95% confidence interval (CI). Continuous outcomes were expressed as weighted mean difference (WMD) or standardized mean difference (SMD) with 95% CI. SMD would be applied in preference if the included studies used different units or rating instruments [19]. *P* < 0.05 was considered to indicate a statistically significant difference.

#### 2.6.2. Assessment of Heterogeneity

The heterogeneity of the included studies was analyzed with the *χ*^2^ test. When *I*^2^ ≤ 50%, a small heterogeneity was considered among the studies, and the fixed effects model was used for data analysis. In case the statistical heterogeneity was *I*^2^ > 50%, the sources of heterogeneity were measured. Subgroup analysis was performed in the presence of clinical heterogeneity, such as the subtypes of ACS, including STEMI, NSTEMI, and UA. In case of significant heterogeneity, the random effects model was considered, or only the descriptive analysis was performed.

#### 2.6.3. Sensitivity Analysis

The studies with high weight and low quality were excluded. Following the comparison of the pooled statistics prior to and following exclusion of these studies, certain differences could be found. Subgroup and sensitivity analyses were also conducted to explore the stability of the results if necessary.

## 3. Results

### 3.1. Study Selection

**Figure 1:**
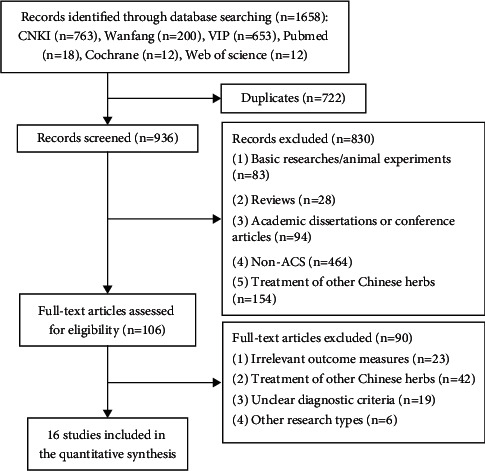
Flow chart for searching and screening of the studies.

A total of 1,658 articles were retrieved from six literature databases. Following the removal of 722 duplicates, 936 potentially relevant articles remained for subsequent assessment. Following evaluation of titles and abstracts, 830 articles were excluded. A total of 90 out of 106 remaining studies were excluded following the investigation of the full texts. Finally, 16 studies [20–35] were included in the meta-analysis. A flow chart (Figure 1) indicated the search process and study selection.

### 3.2. Study Characteristics

A total of 1,658 records were identified and 16 RCTs [20–35] were included, covering 1,171 participants. All the studies were conducted in China and almost all of them were published in Chinese. Only one was published in English. They were published from 2004 to 2020. The sample size varied from 49 to 104 subjects, and the duration of the XFZYD treatment ranged from 1 to 12 weeks. The disease diagnoses of these studies were AMI, STEMI, UNSTEMI, and UA with or without PCI. Further details regarding the characteristics of the included studies are shown in Table 1 and supplementary Table S1.

### 3.3. Risk of Bias and Methodological Quality

**Figure 2:**
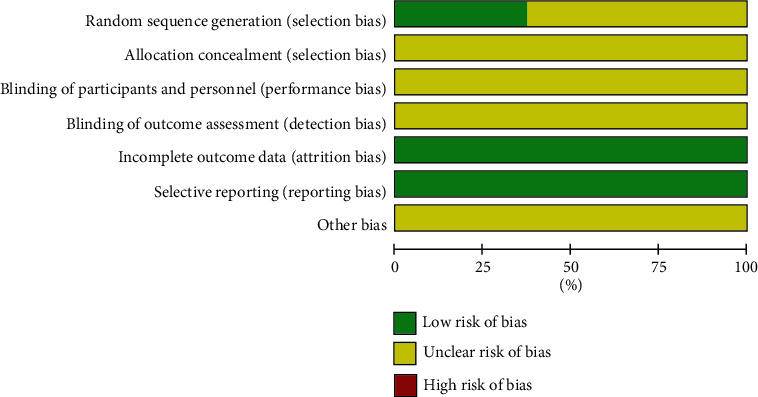
Risk of bias graph.

According to the assessment tools provided by the handbook of Cochrane Collaboration, the included studies displayed methodological bias (Figure 2). All the included studies were described as “randomized” studies; among them, five studies reported using the “random number table” [21, 26, 27, 29, 31], and one study used the toss method [23]. However, the allocation concealment, the blinding of the participants, and the outcome assessment were not clear in any of the studies investigated. The data reported in the results were complete according to the methods used. Therefore, the incomplete outcome data were identified as low risks. Although not all the studies included provided protocols, the expected outcome indicators were reported and the selective reporting bias was defined as low risk. The results indicated that the methodological quality of the 16 literature studies included in this meta-analysis was generally mediocre.

### 3.4. Outcome Measures

#### 3.4.1. Cardiac Functions

**Figure 3:**
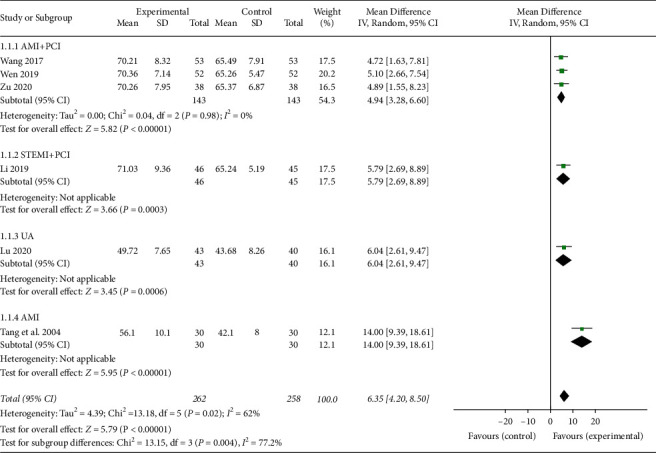
The LVEF of the included studies.

In total, six studies [20–23, 25, 35] reported the outcome of LVEF from the ECG. Three studies [20, 22, 25] included AMI patients with PCI, one study [21] included only UA patients, one study [35] included AMI patients, and one study [23] explicitly included STEMI patients with PCI. Subgroup meta-analysis was performed due to the high total heterogeneity (*I*^2^ = 62%), and the random effects model was used in the meta-analysis. As shown in Figure 3, the combination of XFZYD with RT therapy performed better in improving LVEF (MD 6.35, 95% CI 4.20 to 8.50; *P* < 0.00001; *I*^2^ = 62%). Therefore, it could be used to maintain the cardiac function, and the heterogeneity among AMI with the PCI subgroup was reduced to 0%.

**Figure 4:**
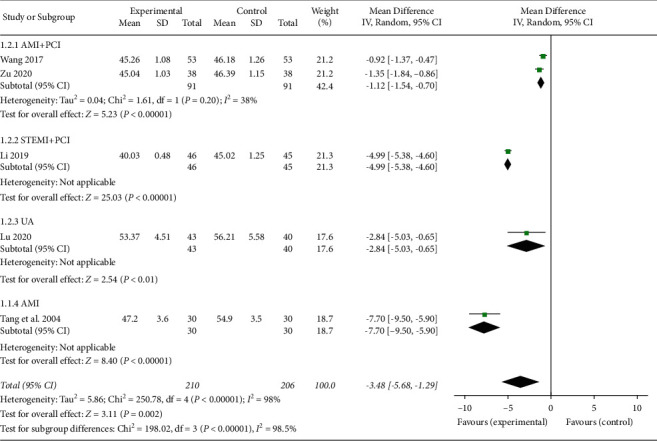
The LVEDD of the included studies.

LVEDD and LVESD were also collected from the echocardiography data and were used to indicate cardiac functions. Among the included studies, five studies [20, 21, 23, 25, 35] reported LVEDD and three [23, 25, 35] reported LVESD. As shown in Figure 4, five studies demonstrated a high heterogeneity (*I*^2^ = 98%), and the subgroup meta-analysis was applied to identify the AMI patients with PCI [20, 25], the STEMI patients with PCI [23], the UA patients [21], and the AMI patients [35]. The results indicated that the combination of XFZYD with RT therapy could decrease LVEDD (MD −3.48, 95% CI −5.68 to −1.29; *P*=0.002; *I*^2^ = 98%). The subgroup of AMI patients with PCI reduced the heterogeneity to 38% and resulted in a positive result. Following analysis of LVESD, the three studies included AMI patients with PCI [25], STEMI patients with PCI [23], and AMI patients [35], respectively. Therefore, they were not merged in the meta-analysis. Each of the three studies indicated a significant difference in improving LVESD following comparison of XFZYD plus RT with RT (26.19 ± 0.87 vs. 29.03 ± 2.05; 32.4 ± 3.3 vs. 39.5 ± 3.4; 27.96 ± 1.56 vs. 29.28 ± 1.38).

#### 3.4.2. The Endothelial Functions

**Figure 5:**
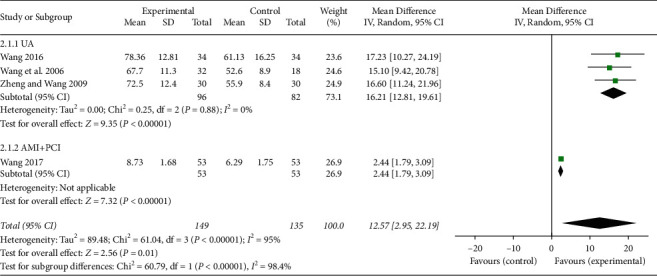
The serum NO levels of the included studies.

In total, four studies [25, 27, 31, 34] reported the serum levels of NO, and three of them included UA patients [27, 31, 34]. One study included AMI patients with PCI [25]. A meta-analysis with a random effects model was conducted in all four studies. The results indicated that the serum levels of NO were significantly increased following the comparison of XFZYD plus RT with RT (MD 12.57, 95% CI 2.95 to 22.19; *P*=0.01; *I*^2^ = 95%). However, the heterogeneity was high and the subgroups were performed in the meta-analysis so as to reduce the heterogeneity to 0% as shown in Figure 5.

**Figure 6:**
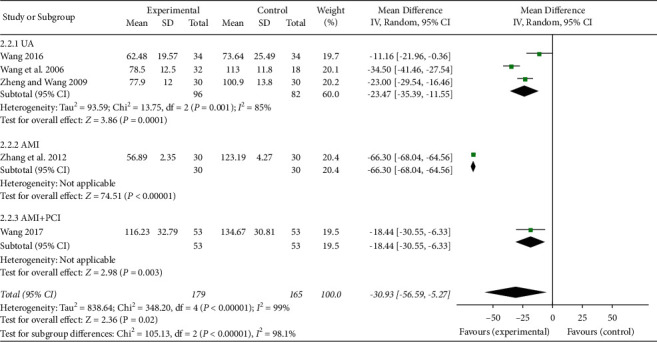
The serum ET-1 levels of the included studies.

The serum levels of ET-1 were also related to the endothelial functions, and a total of five studies [25, 27, 29, 31, 34] reported this index. As shown in Figure 6, a meta-analysis in the random effects model was performed in the five studies. XFZYD plus RT significantly reduced the serum levels of ET-1 compared with those noted for RT (MD −30.93, 95% CI −56.59 to −5.27; *P*=0.02; *I*^2^ = 99%). In the subgroup meta-analysis, the heterogeneity was still high (*I*^2^ = 85%) in the UA group [27, 31, 34] and the outcome was also stable.

**Figure 7:**
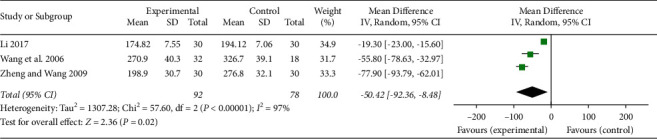
Assessment of the serum levels of ICAM-1 in the included studies.

**Figure 8:**
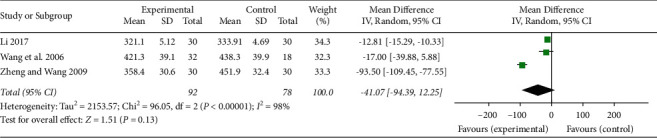
Assessment of the serum levels of VCAM-1 in the included studies.

In total, three studies [26, 31, 34] related to UA patients reported measurement of ICAM-1 and VCAM-1 levels in the serum. These studies were applied in the meta-analysis. The results are shown in Figures 7 and 8. XFZYD plus RT significantly reduced the serum levels of ICAM-1 compared with those of RT alone (MD −50.42, 95% CI −92.36 to -8.48; *P*=0.02; *I*^2^ = 97%), while XFZYD plus RT did not significantly lower the serum levels of VCAM-1 compared with those of RT alone (MD −41.07, 95% CI −94.39 to 12.25; *P*=0.13; *I*^2^ = 98%).

#### 3.4.3. Assessment of CRP and Oxidative Damage Marker Levels

**Figure 9:**
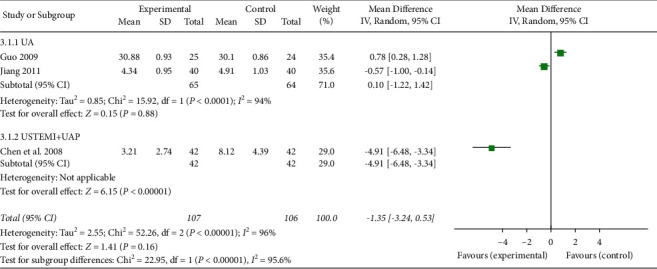
The serum CRP levels of the included studies.

**Figure 10:**
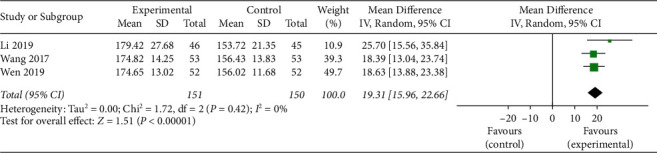
The serum SOD levels of the included studies.

**Figure 11:**
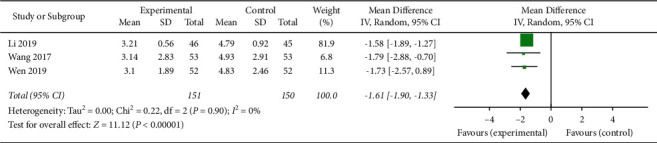
The serum MDA levels of the included studies.

Three studies [30, 32, 33] reported the serum levels of CRP, and three studies [22, 23, 25] reported the determination of both SOD and MDA serum levels. One of the studies [32] indicated that XFZYD plus RT increased the serum levels of CRP compared with those of RT alone (30.88 ± 0.93 vs. 30.1 ± 0.86), which was opposite to the other two studies (3.21 ± 2.74 vs. 8.12 ± 4.39; 4.34 ± 0.95 vs. 4.91 ± 1.03) [30, 33]. In a meta-analysis (Figure 9), the levels of CRP exhibited no significant difference following comparison of XFZYD plus RT with RT (MD −1.35, 95% CI −3.24 to 0.53; *P*=0.16; *I*^2^ = 96%). Moreover, a meta-analysis was conducted to assess the changes in the levels of SOD and MDA (Figures 10 and 11). The results indicated that XFZYD plus RT caused a significant increase in SOD levels (MD 19.31, 95% CI 15.96 to 22.66; *P* < 0.00001; *I*^2^ = 0%), and they also decreased MDA levels (MD −1.61, 95% CI −1.90 to −1.33; *P* < 0.00001; *I*^2^ = 0%) compared with the effects noted following treatment with RT alone. The heterogeneity noted was very low.

#### 3.4.4. The Blood Levels of BNP

**Figure 12:**
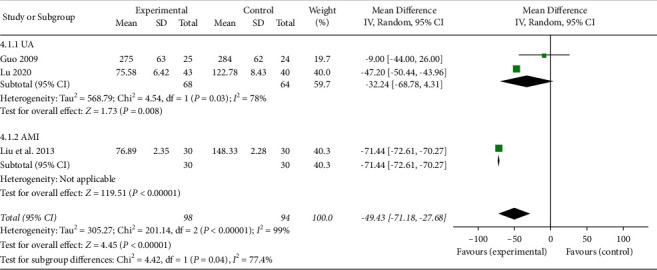
The blood levels of BNP in the included studies.

Three studies [21, 28, 32] reported the blood levels of BNP. As shown in Figure 12, the levels of BNP were significantly different compared with those of RT alone (MD −49.43, 95% CI −71.18 to −27.68; *P* < 0.00001; *I*^2^ = 99%). The subgroups were performed due to the high heterogeneity, and the heterogeneity of the UA group [21, 32] was reduced to 78%.

#### 3.4.5. Myocardial Enzymes

**Figure 13:**
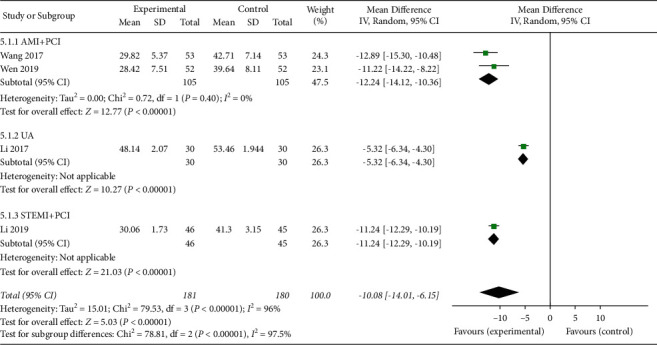
The blood levels of CK-MB in the included studies.

In total, four studies [22, 23, 25, 26] reported the assessment of CK-MB as a cardiac marker, and two of them [22, 25] included AMI patients with PCI, whereas one study [26] included UA patients, and one [23] STEMI patients with PCI. A total of four studies were involved in a meta-analysis (Figure 13). The results indicated that XFZYD plus RT significantly decreased the levels of CK-MB compared with those noted following RT treatment alone (MD −10.08, 95% CI −14.01 to −6.15; *P* < 0.00001; *I*^2^ = 96%). Due to the high heterogeneity, the subgroups of AMI plus PCI, UA, and STEMI plus PCI were used. The heterogeneity in the AMI plus PCI subgroup was very low (*I*^2^ = 0%). One study [26] reported on the changes in the levels of cTnI between the XFZYD plus RT and the RT groups. The results indicated that the XFZYD plus RT group performed better than the RT group alone (0.099 ± 0.019 vs. 0.106 ± 0.038, *P* < 0.05).

#### 3.4.6. ADRs

Two studies [24, 26] on UA reported ADRs in the treatment or control groups. One study [24] reported three mild diarrhea cases in the XFZYD plus RT group and two sinus bradycardia cases in the RT group. The symptoms disappeared following simple symptomatic treatments or the absence of therapies. Another study [26] reported two cases of elevated aminotransferase levels in the control group, whereas no ADRs were noted in the treatment group.

## 4. Discussion

In the present review, 16 studies of ACS were included, of which eight studies reported UA cases, three AMI, three AMI with PCI, one STEMI with PCI, and one NSTE-ACS (NSTEMI + UA). Among these studies, the combination of XFZYD with RT exhibited the potential to improve cardiac and endothelial functions in ACS. The possible mechanisms of the effectiveness of this decoction are discussed below.

LVEF is positively related to the contractility of the myocardium; LVEDD and LVESD represent the diastolic and systolic functions of the left ventricle, respectively [36]. BNP is a quantitative marker of heart failure; CK-MB and cTnI are two sensitive indicators of myocardial damage [37]. The echocardiography results indicated the improvement of cardiac function, and XFZYD aided the amelioration of the levels of LVEF, LVEDD, and LVESD. In addition to these functions, XFZYD has been shown to decrease the blood levels of BNP and the myocardial enzymes (CK-MB and cTnI), representing cardioprotective effects. The cardioprotective function of XFZYD has been verified in various experimental studies. In the rat model of myocardial ischemia-reperfusion injury (MI/RI), XFZYD protected the myocardial ultrastructure [38]. Pretreatment with XFZYD demonstrated protective effects in the myocardial tissues of LPS-induced septic rats by inhibiting cardiomyocyte apoptosis and oxidative stress [39]. XFZYD demonstrated inhibition of myocardial apoptosis by increasing the expression of SIRT1 and inhibiting the expression levels of its downstream proteins p53 and NF-*κ*B in a model of H9c2 rat myocardial cells with oxygen-glucose deprivation [40]. XFZYD was also shown to protect myocardial ischemia rats via the SIRT1-mediated signal transduction pathway [41]. The main components of XFZYD exhibited profound cardioprotective effects [10]. For example, hydroxysafflor yellow A reduced MI/RI injury, suppressed calcium overload in cardiomyocytes, and prevented the induction of their apoptosis [42]. Moreover, chlorogenic acid inhibited activation of the NF-*κ*B and JNK pathways in cardiomyocytes [43].

Endothelial dysfunction is accompanied by the rupture of vulnerable plaques and leads to platelet aggregation and leukocyte adherence [4]. The prominent vasoactive substance NO, released by the endothelium, helps mediate the vasodilation as well as regulates adhesion of leukocytes. NO can be regarded as a protector of cardiovascular endothelial function and vascular inflammation [44]. Endothelial function is also measured by the decreased ET-1 production, which is characterized as a vasoconstrictor [44, 45]. ICAM-1 and VCAM-1 are adhesion molecules. Their expression levels are increased as a result of facilitating leukocyte adhesion and transmigration inside the endothelium, which lead to further inflammatory expansion [46]. Serum CRP levels are also widely regarded as an indicator of inflammation. According to the results, XFZYD can significantly increase the serum levels of NO, and reduce serum ET-1 and ICAM-1 levels, indicating the protective effects on vascular endothelial function. However, XFZYD may not decrease serum VCAM-1 and CRP levels, which may be associated with the sample size of the included study. Therefore, additional research studies are required in the future. The systemic pharmacology of XFZYD in the prevention of atherosclerotic cardiovascular disease (ASCVD) has shown that the mechanism of XFZYD is mainly reflected in the protection of vascular endothelium, prevention of oxidative stress, and inhibition of inflammation; the effective component quercetin was also shown to protect injured endothelial cells and reduce the endothelial inflammatory response (ICAM-1, VCAM-1, and TNF-*α*) induced by LPS *in vitro* [47]. XFZYD has been shown to protect from ischemic necrosis, induce the migration of endothelial progenitor cells, and promote angiogenesis by improving the serum levels of NO [48]. Following XFZYD treatment, the serum levels of ET-1 were decreased and NO levels were released in MI/RI swine [49]. XFZYD can restrain inflammation, platelet aggregation, and help protect reendothelialization after PCI. The indicators reported both from clinical and experimental studies that have shown the efficacy of XFZYD in ASCVD.

SOD is an antioxidant metal enzyme that can catalyze the disproportionation of superoxide anion radicals to produce oxygen and hydrogen peroxide [50]. It plays a vital role in maintaining the balance required (pro-oxidants/antioxidants) to prevent the induction of oxidative stress in the body [50]. In several organisms, free radicals bind to lipids to induce their peroxidation, which results in the production of MDA. The latter can reflect the degree of lipid peroxidation in the body and indirectly reflect the degree of cell damage [51]. Higher SOD and lower MDA levels play inhibitory roles against the development of ACS. The results indicated that XFZYD could upregulate the serum levels of SOD and downregulate the serum levels of MDA, reducing serum oxidative stress levels and improving the function of the endothelial microenvironment. Previous studies have also shown that XFZYD plays an antioxidant role by reducing serum MDA levels and increasing serum SOD levels in CHD patients undergoing PCI surgery [52].

Endothelial dysfunction, oxidative stress, abnormal vasoconstriction, and dilation are crucial in the pathogenesis of atherosclerosis and ACS [53, 54]. XFZYD can improve cardiac function by increasing blood supply to myocardium and can ameliorate coronary microcirculation by improving endothelial function. Future studies can focus on these mechanisms for ACS to uncover more potential drug targets and long-term effects of this formula. Based on these results, the practical applications of XFZYD with conventional therapy can be used in ACS patients as soon as possible.

The limitations of this systematic review can be summarized in the following three aspects: First of all, the number of studies included in the meta-analysis was considerably small, and only six studies were involved in the meta-analysis of the primary outcome (LVEF), which could not be used for analyzing the publication bias. Moreover, the heterogeneity among all the ACS subtypes was too high in the meta-analysis. Therefore, subgroup analysis was conducted according to the ACS subtypes of STEMI, USTEMI, and UA, with or without PCI. Lower heterogeneity was detected, whereas a smaller number of studies were involved in the subgroup analysis, which may lead to unstable results. Finally, almost all the included studies were published in Chinese, while only one was published in English, and none of them reported the allocation concealment and blinding method, which augmented the risk of bias in RCTs.

## 5. Conclusions

In summary, the present analysis demonstrated that XFZYD can be used as a representative Chinese prescription, which may possess significant clinical applications for improving cardiac and endothelial functions and the LVEF, LVEDD, LVESD, NO, ET-1, and ICAM-1 of ACS patients when combined with RT. Moreover, it may also ameliorate the levels of oxidative stress (SOD and MDA), BNP, CK-MB, and cTnI. No serious ADRs were noted. However, XFZYD may not improve the blood levels of VCAM-1 and CRP. On the basis of these results, we prefer to confirm the amelioration of cardiac and endothelial functions of XFZYD in ACS. However, considering the mediocre methodological quality and a small number of included studies, it is deduced that multicenter, large-scale, and strictly designed trials are required to confirm these findings. This systematic review has provided evidence for the clinical efficacy of XFZYD combined with RT in treating patients with ACS.

## Figures and Tables

**Table 1:** Characteristics of the included studies.

Study ID	Sample size (T/C)	Type of disease	Age (T/C)	Baseline	Interventions		Duration	Outcome measures
					Treatment group	Control group		
Lu (2020) [21]	43/40	UA	62.57 ± 8.42/63.39 ± 8.90	C	XFZYD + amiodarone	Amiodarone	4w	①②⑪
Zu (2020) [20]	38/38	AMI + PCI	59.68 ± 8.46/60.16 ± 8.26	C	XFZYD + RT	RT	8w	①②
Li (2019) [23]	46/45	STEMI + PCI	59.03 ± 3.62/58.19 ± 3.86	C	XFZYD + RT	RT	1w	①②③⑨⑩⑫
Wen (2019) [22]	52/52	AMI + PCI	60.30 ± 4.85/60.25 ± 4.87	C	XFZYD + RT	RT	4w	①⑨⑩⑫
Liu and Liu (2018) [24]	40/40	UA	NA/NA	C	XFZYD + RT	RT	4w	⑭
Li (2017) [26]	30/30	UA	57.9 ± 8.3/58.2 ± 8.5	C	XFZYD + RT	RT	2w	⑥⑦⑫⑬⑭
Wang (2017) [25]	53/53	AMI + PCI	57.36 ± 8.24/59.02 ± 9.78	C	XFZYD + RT	RT	2w	①②③④⑤⑨⑩⑫
Wang (2016) [27]	34/34	UA	NA/NA	C	XFZYD + RT	RT	8w	④⑤
Liu et al. (2013) [28]	30/30	AMI	NA/NA	C	XFZYD + RT	RT	2w	⑪
Zhang et al. (2012) [29]	30/30	AMI	61 ± 7/59 ± 8	C	XFZYD + RT	RT	2w	⑤
Jiang (2011) [30]	40/40	UA	NA/NA	C	XFZYD + RT	RT	4w	⑧
Guo (2009) [32]	25/24	UA	NA/NA	C	XFZYD + RT	RT	2w	⑧⑪
Zheng and Wang (2009) [31]	30/30	UA	64.8 ± 7.9/65.2 ± 7.4	C	XFZYD + RT	RT	8w	④⑤⑥⑦
Chen et al. (2008) [33]	42/42	USTEMI + UA	62.83 ± 10.14/64.83 ± 10.75	C	XFZYD + RT	RT	8w	⑧
Wang et al. (2006) [34]	32/18	UA	65.4 ± 7.6/65.3 ± 7.8	C	XFZYD + RT	RT	8w	④⑤⑥⑦
Tang et al. (2004) [35]	30/30	AMI	NA/NA	C	XFZYD + RT	RT	12w	①②③

① LVEF; ② LVEDD; ③ LVESD; ④ NO; ⑤ ET-1; ⑥ ICAM-1; ⑦ VCAM-1; ⑧ CRP; ⑨ SOD; ⑩ MDA; ⑪ BNP; ⑫ CK-MB; ⑬ cTnI; ⑭ ADRs. The numerical values of age are presented as mean value ± standard deviation in the treatment group (T) and the control group (C). Abbreviations: ADRs, adverse drug reactions; AMI, actue myocardial infarction; BNP, brain natriuretic peptide; C, consistent; CK-MB, creatine kinase-MB; CRP, C-reactive protein; cTnI, cardiac troponin I; ET-1, endothelin-1; ICAM-1, intercellular adhesion molecule-1; LVEF, left ventricular ejection fraction; LVEDD, left ventricular end-diastolic diameter; LVESD, left ventricular end-systolic diameter; MDA, malondialdehyde; NA, not available; NO, nitric oxide; NSTEMI, non-ST-segment elevation myocardial infarction; PCI, percutaneous coronary intervention; RT, routine treatment; SOD, superoxide dismutase; STEMI, ST-segment elevation myocardial infarction; T/C, treatment group/control group; UA, unstable angina; VCAM-1, vascular cell adhesion molecule-1; w, week; XFZYD, Xue-Fu-Zhu-Yu decoction.

## Data Availability

All the data generated or analyzed during this study are included in this published article and its additional files.

## Abbreviations

ACS: Acute coronary syndrome
ADRs: Adverse drug reactions
AMI: Actue myocardial infarction
ASCVD: Atherosclerotic cardiovascular disease
BNP: Brain natriuretic peptide
CK-MB: Creatine kinase-MB
CHD: Coronary heart disease
CI: Confidence interval
CRP: C-reactive protein
cTnI: Cardiac troponin I
ECG: Electrocardiogram
ET-1: Endothelin-1
ICAM-1: Intercellular adhesion molecule-1
ICD-10: International classification of diseases10th revision
LVEF: Left ventricular ejection fraction
LVEDD: Left ventricular end-diastolic diameter
LVESD: Left ventricular end-systolic diameter
MDA: Malondialdehyde
MI: Myocardial infarction
MI/RI: Myocardial ischemia-reperfusion injury
NO: Nitric oxide
NSTEMI: Non-ST-segment elevation myocardial infarction
PCI: Percutaneous coronary intervention
RCTs: Randomized controlled trials
RT: Routine treatment
SMD: Standardized mean difference
SOD: Superoxide dismutase
STEMI: ST-segment elevation myocardial infarction
TCM: Traditional Chinese medicine
UA: Unstable angina
VCAM-1: Vascular cell adhesion molecule-1
WMD: Weighted mean difference
XFZYD: Xue-Fu-Zhu-Yu decoction.

## Supplementary Materials

Supplementary Materials Table S1: other characteristics of the included studies.
