# Supplementary material for: The Improvement of Cardiac and Endothelial Functions of Xue-Fu-Zhu-Yu Decoction for Patients with Acute Coronary Syndrome: A Meta-Analysis of Randomized Controlled Trials
Source: Evid Based Complement Alternat Med. 2022 Feb 10;2022:2671343. doi: 10.1155/2022/2671343 (PMC8853789; doi:10.1155/2022/2671343)
Supplement: Supplementary Materials — Table S1: other characteristics of the included studies. [file 2671343.f1.docx]

Supplementary *table S1*. Other characteristics of the included studies.

| Study ID | Routine treatment in detail | Dose of XFZYD |
| --- | --- | --- |
| Lu (2020)[21] | Amiodarone | ①9g; ②9g; ③12g; ④9g; ⑤6g; ⑥6g; ⑦6g; ⑧3g; ⑨4.5g; ⑩4.5g; ⑪9g; water decoction, twice a day. |
| Zu (2020)[20] | Aspirin, clopidogrel | ①9g; ②9g; ③12g; ④9g; ⑤3g; ⑥6g; ⑦6g; ⑧3g; ⑨5g; ⑩6g; ⑪9g; water decoction, twice a day. |
| Li (2019)[23][Li, 2019 #49] | Atorvastatin, aspirin, antihypertensive treatment and hypoglycemic agent | ①15g; ②15g; ③10g; ④12g; ⑤10g; ⑥6g; ⑦12g; ⑧6g; ⑨10g; ⑩6g; ⑪10g; water decoction, twice a day. |
| Wen (2019)[22] | Pravastatin, aspirin, clopidogrel, Low-molecular-weight heparin | ①9g; ②9g; ③12g; ④9g; ⑤3g; ⑥6g; ⑦6g; ⑧3g; ⑨5g; ⑩6g; ⑪9g; water decoction, twice a day. |
| Liu and Liu (2018)[24] | Aspirin, clopidogrel, atorvastatin, nitrate esters, antihypertensive treatment and hypoglycemic agent | XFZY granule (approval No. Z20050016), 6g, three times a day. |
| Li (2017)[26] | Not specified | ①10g; ②15g; ③12g; ④10g; ⑤6g; ⑥15g; ⑦15g; ⑧3g; ⑨10g; ⑩15g; ⑪10g; water decoction, twice a day. |
| Wang (2017)[25] | Pravastatin, aspirin, clopidogrel, Low-molecular-weight heparin | ①9g; ②9g; ③12g; ④9g; ⑤3g; ⑥6g; ⑦6g; ⑧3g; ⑨5g; ⑩6g; ⑪9g; water decoction, twice a day. |
| Wang (2016)[27] | Isosorbide mononitrate, aspirin, atorvastatin, amlodipine besylate, metoprolol tartrate | ①10g; ②10g; ③12g; ④10g; ⑤6g; ⑥6g; ⑦15g; ⑧6g; ⑨6g; ⑩6g; ⑪10g; water decoction, twice a day. |
| Liu et al. (2013)[28] | Aspirin, betaloc, nitroglycerin, captopril, heparin, etc. | ①9g; ②9g; ③12g; ④9g; ⑤6g; ⑥9g; ⑦6g; ⑧3g; ⑨5g; ⑩5g; ⑪9g; water decoction, twice a day. |
| Zhang et al. (2012)[29] | Aspirin, betaloc, nitroglycerin, captopril, heparin, etc. | ①9g; ②9g; ③12g; ④9g; ⑤6g; ⑥9g; ⑦6g; ⑧3g; ⑨5g; ⑩5g; ⑪9g; water decoction, twice a day. |
| Jiang (2011)[30] | Not specified | XFZY capsule (approval No. Z12020223), 2.4g, twice a day. |
| Guo (2009)[32] | Isosorbide mononitrate, simvastatin | XFZY capsule (approval No. Z12020223), 2.4g, twice a day. |
| Zheng and Wang (2009)[31] | Isosorbide mononitrate, nitroglycerin, nifedipine, metoprolol, aspirin | XFZY capsule (batch No. 040619), 2.4g, twice a day. |
| Chen et al. (2008)[33] | Not specified | ①10g; ②10g; ③10g; ④10g; ⑤3g; ⑥6g; ⑦10g; ⑧6g; ⑨10g; ⑩6g; ⑪10g; water decoction, twice a day. |
| Wang et al. (2006)[34] | Not specified | Unspecified dose |
| Tang et al. (2004)[35] | Diazepam, aspirin, bisoprolol, captopril, isosorbide mononitrate | XFZY oral liquid (batch No. 000112) 10ml, three times a day. |

①*Angelicae Sinensis* (Oliv.), Diels (Danggui); ②*Rehmannia glutinosa* Libosch. (Dihuang); ③*Prunus persica* (L.) Batsch (Taoren);④*Carthamus tinctorius* L. (Honghua); ⑤*Glycyrrhiza uralensis* Fisch. (Gancao); ⑥Aurantii Fructus (Zhiqiao); ⑦*Paeonia lactiflora* Pall. (Chishao); ⑧*Bupleurum chinense* DC. (Chaihu); ⑨*Ligusticumi chuanxiong* Hort. (Chuanxiong); ⑩*Platycodon grandiflorum* (Jacq.) A. DC. (Jiegeng); ⑪*Achyranthes bidentata* Bl. (Niuxi).
